# The Great Imitator Strikes Again: Syphilis Presenting as “Tongue Changing Colors”

**DOI:** 10.1155/2016/1607583

**Published:** 2016-01-20

**Authors:** Jessica Swanson, Janna Welch

**Affiliations:** University of Texas Dell School of Medicine, Austin, TX 78701, USA

## Abstract

Syphilis is known as the great imitator, making its diagnosis in the emergency department difficult. A 29-year-old male presented with the chief complaint of “my tongue is changing colors.” A syphilis rapid plasma reagin (RPR) test resulted as positive. In primary syphilis, the chancre is the characteristic lesion. While chancres are frequently found on the external genitalia or anus, extragenital chancres arise in 2% of patients. With oral involvement, the chancre is commonly found on the lip or tongue. The patient was treated for secondary syphilis with 2.4 million units of long acting penicillin intramuscularly. On follow-up a month later, the patient's symptoms had resolved.

## 1. Case Presentation

A 29-year-old male presented to the emergency department with the chief complaint of “my tongue is changing colors.” The symptoms had been present for the past week. He denied any pain or history of similar symptoms in the past. On review of systems, his only other complaint was arthralgias, primarily located in his hips and knees bilaterally. Of note, he denied any recent rashes, weight loss, hair loss, or genital lesions. His past medical history was significant only for a chlamydia infection five years previously, which had been treated. On social history, he denied any alcohol or illicit drug use, including any intravenous drugs. He did endorse having sex with men, and his most recent new sexual partner was 2 months ago.

**Figure 1:**
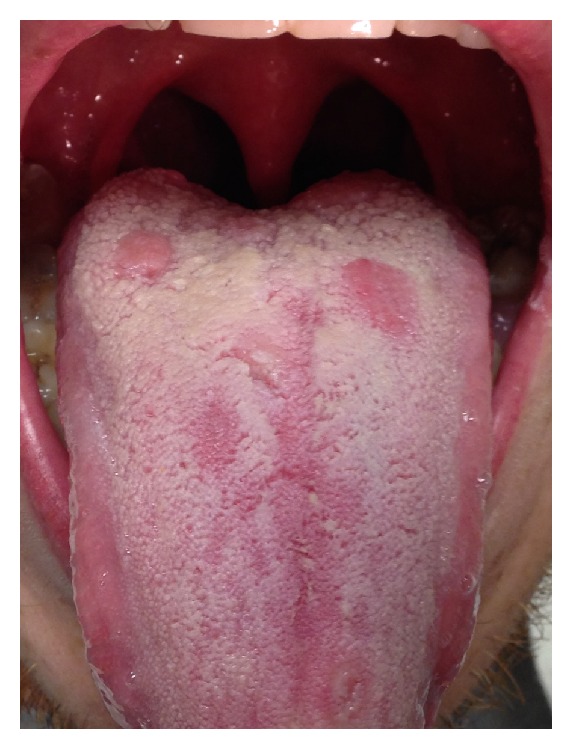


On physical exam, he was found to be afebrile with normal vital signs. Overall, he was a thin, well appearing male. Head and neck exam revealed three shallow, nonpainful erosions covered with a grayish membrane on the posterior aspect of his tongue (Figure 1). There was no cervical lymphadenopathy. Exam of his hips and knees was unimpressive; there was no swelling, redness, or pain with palpation or range of motion. There were no lesions or rashes on the skin.

There is a broad differential diagnosis for pigmented macules of the oropharynx, which includes autoimmune, viral, mycotic causes as well as hereditary syndromes, heavy metal ingestion, cigarette use, and medication related cause. In addition to taking a thorough history, we performed basic screening complete blood count, metabolic panel, and erythrocyte sedimentation rate, which were normal. We did not order a lead level but kept heavy metal in the differential in cases the syphilis testing was negative. A screening syphilis qualitative RPR test was ordered, which came back positive. The confirmatory microhemagglutination assay, (MTA-TPS) which recognizes treponema to rule out false positives, also indicated syphilis. The patient was treated for secondary syphilis with 2.4 million units of long acting penicillin intramuscularly. Of note, the patient declined HIV testing in the emergency department. On follow-up a month later, the patient's symptoms had resolved.

## 2. Discussion

Syphilis, known as the great imitator, can present in a variety of ways, making its diagnosis in the emergency department difficult. As emergency medicine practitioners, we must keep syphilis on our differential list as its incidence continues to rise, particularly among men who have sex with men (MSM). In 2013, there were 56,471 new reported syphilis cases in the US. According to the Center for Disease Control, between 2012 and 2013, the number of reported primary and secondary syphilis cases increased by 10.9%, with 75% of these cases in MSM. Men aged 20–29 had the highest incidence of primary and secondary syphilis, followed by women 20–24 years old (CDC).

Characteristic skin findings are found in all stages of syphilis. Oral manifestations are significantly less common than those of the skin but can be seen in all stages of syphilis (Table 1). Only one case (from 1978) was found in the author's literature search in which a patient with secondary syphilis presented solely with complaints of changes to the tongue [1]. A PubMed literature review performed by Leuci et al. looked at the oral involvement of syphilis over a 61-year time period. Their literature review found only 34 patients with reports of oral involvement. In addition, they also reported a retrospective, multicenter case series of 12 patients that had presented with oral manifestations of syphilis in all stages of disease. Of those with secondary syphilis, most had some other manifestation of the disease aside from oral involvement.

In primary syphilis, the chancre is the characteristic lesion. It develops at the site of inoculation, beginning as a papule that progresses to ulceration. Chancres are generally painless, solitary lesions, although they can be multiple. While chancres are most frequently found on the external genitalia or anus, extragenital chancres arise in 2% of patients [2]. Of the extragenital sites, the mouth is the site in 40–70% of [3]. When the mouth is involved, the chancre is most commonly found on the lip and occasionally the tongue. Rarely, the pharynx or tonsils may be involved. The upper lip is more commonly affected in males and the lower lip in females. Cervical lymphadenopathy generally accompanies the chancre [4]. Regardless of location, the chancre typically regresses, regardless of treatment, after 2–8 weeks [5].

Secondary syphilis is characterized by a variety of nonspecific, flu-like symptoms, including fever, malaise, headache, sore throat, and arthralgias. A disseminated, symmetric rash occurs in 75% of patients [3]. The morphology of the rash varies greatly, from macular to maculopapular to nodular. It characteristically involves the palms of the hands and soles of the feet. Additional symptoms may include ocular manifestations, condyloma lata, hepatitis, arthritis, and neurologic involvement [5].

Approximately, 30% of patients with secondary syphilis have involvement of the oral cavity. However, oral findings are rarely the only manifestation [6]. The primary oral manifestations of secondary syphilis are mucus patches (as seen in this patient) and maculopapular lesions, although nodules may be found as well. The mucous patches are typically slightly raised and covered with a grayish white pseudomembrane. Macular lesions are generally found on the hard palate, while mucus patches are most commonly found on, but not limited to, the tongue [6].

Tertiary syphilis can present with neurosyphilis or cardiovascular syphilis or as gummatous syphilis. The gumma is granulomatous lesion, often found on the skin, bone, or liver. However, gummas can involve any organ [7]. In the oral cavity, it is most commonly seen as a swelling on the tongue or hard palate, which eventually ulcerates. Gumma complications include bone erosion, palatal perforation, and oronasal fistulas [8].

When a diagnosis of syphilis is suspected, serological testing should be performed. Nontreponemal and treponemal tests are the standard for diagnosing syphilis in the US in all stages of the disease [9]. Generally, nontreponemal tests are performed first, the most common of which are the VDRL or RPR tests. These tests become positive 6 weeks after exposure and 1–4 weeks after appearance of the primary lesion. In the case of a positive test result, it should be confirmed with a treponemal test [8]. As received by the patient in this case, 2.4 million units of benzathine penicillin is the treatment of choice for patients diagnosed with primary, secondary, or early latent syphilis [10].

When a diagnosis of syphilis is made, it is important to consider testing for HIV as well. While our patient declined an HIV test in the emergency department, we educated him on how to obtain HIV testing should he change his mind. As of 2002, incidence of syphilis in HIV infected patients has been reported as 77 times higher when compared to the general population [11]. Syphilis appears to increase the transmission of HIV due to local immunological and bacteriological reactions that occur at the site of chancre formation (CDC). In patients coinfected with HIV, syphilis may present even more subtly than in non-HIV patients. Finally, syphilis serology tests may result in false negatives in HIV infected patients [5].

## 3. Conclusion

Oftentimes, due to issues with access to medical care, the emergency physician must also take on the role of primary care doctor. Thus, it is important to keep a wide differential diagnosis, even with seemingly benign complaints. Syphilis, the great imitator, should remain at the back of our minds. A detailed history, with an emphasis on sexual history, can aid in making the diagnosis. In patients diagnosed with syphilis, HIV testing should be strongly encouraged, as the two diseases are often cotransmitted.

## Figures and Tables

**Table 1:** The oral manifestations of syphilis.

Disease stage	Oral manifestations

Primary syphilis	Chancre: painless or painful

Secondary syphilis	Mucosal patches
	Ulcerations: solitary or multiple
	Leukoplakia-like plaques
	Maculopapular lesions
	Aphthous lesions
	Pseudomembranous lesions
	Condyloma lata

Tertiary syphilis	Gumma
	Atrophic glossitis
	Syphilitic leukoplakia
